# In Vitro Evaluation of Antileishmanial Activity of *Commiphora myrrha* Essential Oil Nanoliposome

**DOI:** 10.1002/vms3.70860

**Published:** 2026-03-31

**Authors:** Zahra Akbari, Mohammad Reza Youssefi, Mohaddeseh Abouhosseini Tabari

**Affiliations:** ^1^ Graduate Student of Veterinary Medicine Islamic Azad University, Babol Branch Babol Iran; ^2^ Department of Veterinary Parasitology Islamic Azad University, Babol Branch Babol Iran; ^3^ Department of Veterinary Biomedical Sciences Shreiber School of Veterinary Medicine New Jersey USA

**Keywords:** antileishmania, *Commiphora myrrha*, essential oil, nanoliposome

## Abstract

**Background/Objective:**

Leishmaniasis poses a significant public health challenge worldwide. Nanotechnology has emerged as a promising avenue in parasitology, offering novel strategies for diagnosing, treating, and preventing diseases like leishmaniasis. Hence, the current study investigates the in vitro efficacy of nanoliposomal formulations containing *Commiphora myrrha* (*C. myrrha*) essential oil in combating *Leishmania tropica* (MHOM/IR/99/YAZ1).

**Methods:**

*Commiphora myrrha* essential oil and nanoliposomes were prepared in RPMI‐1640 medium at concentrations of 1, 2, 5 and 10 µg/dL. Promastigotes were exposed to these compounds for 24 h, and viability was assessed using an MTT (3‐(4,5‐dimethylthiazol‐2‐yl)‐2,5‐diphenyltetrazolium bromide) assay.

**Results:**

Dynamic light scattering analysis revealed nanoliposomes of *C. myrrha* essential oil to be approximately 122.3 nm in size with a polydispersity index of 0.852, indicating nanoscale formulation and good particle dispersion. Significantly higher mortality rates were observed in promastigotes treated with nanoliposomes compared to those treated with *C. myrrha* essential oil alone (*p* < 0.005). Glucantime treatment demonstrated the highest mortality rate, significantly differing from *C. myrrha* essential oil and nanoliposome treatments (*p* < 0.001). The 50% lethal concentration (LC50) for *C. myrrha* essential oil nanoliposomes was determined to be 15.30 µg/mL (95% confidence interval: 10.70–28.04 µg/mL).

**Conclusion:**

The results of the present study are promising, suggesting that *C. myrrha* essential oil nanoliposomes could play a significant role in the fight against *L. tropica*. Further in vivo studies with different doses are recommended.

## Graphical abstract

This study investigates the efficacy of nanoliposomal formulations containing myrrh essential oil in combating *Leishmania* protozoa in laboratory settings.

Myrrh essential oil and nanoliposomes were prepared in RPMI‐1640 medium at concentrations of 1, 2, 5 and 10 µg/dL. Promastigotes were exposed to these compounds for 24 h, and viability was assessed using an MTT assay.

Dynamic Light Scattering analysis revealed nanoliposomes of myrrh essential oil to be approximately 122.3 nm in size with a polydispersity index of 0.852, indicating nanoscale formulation and good particle dispersion. Significantly higher mortality rates were observed in promastigotes treated with nanoliposomes compared to those treated with myrrh essential oil alone (*p* < 0.005). Glucantime treatment demonstrated the highest mortality rate, significantly differing from essential oil and nanoliposome treatments (*p* < 0.001).

The findings suggest that myrrh essential oil nanoliposomes exhibit promising anti‐*Leishmania* activity under controlled laboratory conditions.

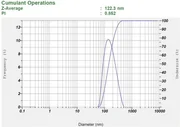

## 1 Introduction

Leishmaniasis, caused by the protozoan parasite *Leishmania*, poses a significant public health challenge worldwide, particularly in regions with limited resources and inadequate healthcare infrastructure (Saini et al. 2022; Wamai et al. 2020). With millions affected annually and up to 1.2 million new cases reported each year, this disease manifests in various forms, ranging from skin lesions to internal organ involvement, each presenting unique challenges in treatment and management (Thakur et al. 2020). Despite efforts to control its spread, obstacles like drug resistance, limited treatment options and ineffective vector control persist, underscoring the urgent need for innovative therapeutic approaches (Coutinho De Oliveira et al. 2020; Mazire et al. 2022).

Leishmaniasis requires a multifaceted approach to treatment that varies depending on the type and severity of the disease (Assolini et al. 2022; Gutiérrez et al. 2016). There are several conventional treatments for leishmaniasis, most of which involve antileishmanial agents such as pentavalent antimonials, miltefosine and other antibiotics (Roatt et al. 2020; Sundar and Chakravarty 2017). There are often side effects associated with these drugs, such as damage to the liver and kidneys. Among the most recent treatments for this disease are antiviral drugs and immunological therapies (Chakravarty and Sundar 2019; Sasidharan and Saudagar 2021; Sundar et al. 2019). However, these methods still need to be fully validated and require additional research to be fully effective. There are many challenges in treating leishmaniasis, including drug resistance, remote medicine inaccessibility and the high cost of medications (de Vries and Schallig 2022). In some cases, these challenges can hurt treatment effectiveness.

Various medicinal plants have been regarded as an effective and natural way to treat zoonotic diseases since herbal medicines are recognized as a natural and potential tool in the fight against zoonotic diseases such as leishmaniasis (Bahmani et al. 2015; Oryan 2015). As many plants contain compounds that are antimicrobial, antiviral and anti‐inflammatory, they prove to be effective in treating and preventing zoonotic diseases, as well as preventing them from spreading (Bekhit et al. 2018; Et‐Touys et al. 2017; Gharirvand Eskandari et al. 2020). For example, turmeric and garlic are being studied for their antimicrobial properties in improving the immune system (Oyinlola et al. 2024; Prajapati et al. 2021). Although there are some major challenges ahead, these include insufficient scientific research, prescription dosage risks and the uncontrolled quality of herbal products.

*Commiphora myrrha* essential oil, derived from the resin of Commiphora trees, has garnered attention in medical research due to its diverse medicinal properties, including antimicrobial activity, anti‐inflammatory effects and wound‐healing capabilities (Althurwi et al. 2022; Gebrehiwot et al. 2015). In leishmaniasis, *C. myrrha* essential oil has demonstrated efficacy against *Leishmania* parasites, attributed to its potent antimicrobial components (Awad et al. 2021). However, challenges exist due to the oil's limited solubility and rapid degradation, hindering its therapeutic utility.

Nanotechnology has emerged as a promising avenue in parasitology, offering novel strategies for diagnosing (Sadr, Hajjafari, et al. 2025), treating and preventing diseases like leishmaniasis (Babaei et al. 2024; Sadr, Lotfalizadeh, et al. 2023; Tiwari et al. 2023). Nanomaterials’ unique properties, including their size, high surface area‐to‐volume ratio, and customizable surface chemistry, enable targeted drug delivery, enhancing bioavailability and therapeutic efficacy (Sadr, Poorjafari Jafroodi, et al. 2023). In leishmaniasis treatment, nanotechnology interventions hold promise in overcoming challenges such as low drug solubility, systemic toxicity and drug resistance (Kammona and Tsanaktsidou 2021).

Nanotechnology offers a solution to these challenges by enabling the encapsulation of essential oils within nanocarriers such as liposomes (Saeed et al. 2022). This approach enhances the stability and bioavailability of the oil while facilitating targeted delivery to infection sites (Javed et al. 2020; Saka and Chella 2021). By harnessing the synergies between essential oils and nanotechnology, innovative formulations like nanoliposomes hold promise for developing effective treatments for leishmaniasis and other parasitic diseases (Dewi et al. 2022). This research aims to investigate the effectiveness of nanoliposomes containing *C. myrrha* essential oil in combating *Leishmania tropica* parasites in laboratory settings, offering a potential avenue for safe and affordable treatment options in regions endemic to these diseases.

## 2 Methods and Materials

### 2.1 Preparation of *Commiphora myrrha* Essential Oil Nanoliposomes

*Commiphora myrrha* essential oil, sourced from the myrrh plant through hydrodistillation, served as the primary ingredient in the formulation. Other components, including Tween 80 (polyoxyethylene sorbitan), phosphatidylcholine and chloroform, were procured from Sigma Aldrich (Germany). A phosphate buffer saline (PBS) solution comprising sodium chloride, potassium chloride, dibasic sodium phosphate and potassium dihydrogen phosphate was prepared from Merck (Germany).

Creating nanoliposomes commenced by combining 1 g of *C. myrrha* oil with a solution containing 0.5 g of phosphatidylcholine and 0.3 g of Tween 80 in 30 mL of chloroform. This mixture was stirred for an hour at a temperature of 55°C and at a stirring speed of 1800 rpm using a stirrer. Subsequently, the resulting blend was subjected to layer‐by‐layer drying on a rotary evaporator under vacuum conditions at 60°C. The evaporator's product was then collected and immersed for 24 h in a 60‐mL phosphate buffer solution adjusted to a pH level of 7.4, undergoing rapid rotation to ensure homogenous dispersion.

The mixture underwent 1 h of ultrasonic treatment in an ultrasonic bath to further enhance particle dispersion. The resulting product underwent rigorous testing to confirm its nanometre‐scale dimensions. Dynamic light scattering (DLS) analysis was utilized to assess particle size distribution and determine the zeta potential, ensuring the quality and uniformity of the nanoliposome formulation.

### 2.2 Cultivation of Parasites

Promastigotes of *L. tropica* were maintained in a controlled environment at a temperature ranging between 25°C and 26°C. They were cultured in a medium supplemented with 10% foetal bovine serum, streptomycin (100 µm/mL), and penicillin (100 U/mL) to support their growth and viability. To prevent genetic drift and maintain consistency, the promastigotes were passaged every 3–4 days, ensuring they remained within three passages during their time in the laboratory.

Before treatment, the promastigotes were diluted to 1 × 10^6^/mL in the culture medium. This standardized concentration ensured uniformity across experimental conditions and facilitated accurate assessment of treatment effects on parasite viability and proliferation. Treatment concentrations were then added to the parasite‐containing culture medium, allowing for the evaluation of the efficacy of the interventions against *L. tropica* promastigotes.

### 2.3 Assessment of Antileishmanial Activity

*Commiphora myrrha* essential oil and *C. myrrha* nanoliposomes were prepared in RPMI 1640 medium at final concentrations of 1, 2, 5 and 10 µg/dL to evaluate their activity against *L. tropica* promastigotes. Glucantime served as the control at varying concentrations (mL/UN). Each assay plate well received 100 µL of promastigotes at a concentration of 1.0 × 10^4^ cells per well, with three wells left untreated as negative controls.

Following the addition of the respective compounds, the viability of the promastigotes was assessed using an MTT (3‐(4,5‐dimethylthiazol‐2‐yl)‐2,5‐diphenyltetrazolium bromide) assay after a 24‐h treatment period. MTT solution (pH 7.4) was added to each well and incubated overnight in darkness at approximately 25°C. Subsequently, a solution containing 50% isopropanol and 10% sodium dodecyl sulphate (SDS) was added, followed by incubation at 37°C for 5 h. Absorbance readings of all samples were then taken at a wavelength of 540 nm using an enzyme‐linked immunosorbent assay (ELISA) reader.

Each experiment was repeated three times to confirm accuracy and consistency, ensuring the reliability of the results. This rigorous approach helped validate the efficacy of the tested compounds against *L. tropica* promastigotes and provided robust data for analysis.

### 2.4 Statistical Analysis

The analysis was performed using IBM SPSS version 26 software (Chicago, USA), ensuring robust and reliable statistical outcomes for interpretation. The statistical analysis began with assessing the normality of the data using the Smirnov–Kolmogorov test, which confirmed its acceptance. Given the non‐normal distribution of the data, the Wallis–Kruskal test was employed to compare differences among various treatments and doses. The Mann–Whitney *U* test also compared oil, nanoliposome and glucantime treatments pairwise. Probit regression analysis determined the lethal concentrations (LC) at 50% and 90% (LC50 and LC90). This method allowed for estimating the concentration at which the respective treatments killed 50% and 90% of the promastigotes. A significance level of *p* ≤ 0.05 was considered statistically significant for all tests conducted.

## 3 Results

### 3.1 DLS and Zeta Analysis

**FIGURE 1:**
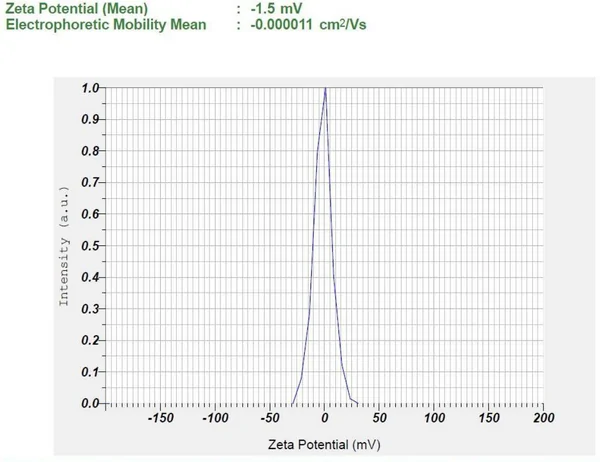
Zeta potential analysis of *Commiphora myrrha* nanoliposomes.

The tests to characterize the size and dispersion of the *C. myrrha* essential oil nanoliposome formulation confirmed its nanometre dimensions. DLS analysis revealed that the *C. myrrha* essential oil nanoliposomes have an average size of approximately 122.3 nm, with a polydispersity index of 0.852. These results indicate uniformity in particle size distribution, reflecting formulation at the nanoscale and excellent particle dispersion (Figure 1).

**FIGURE 2:**
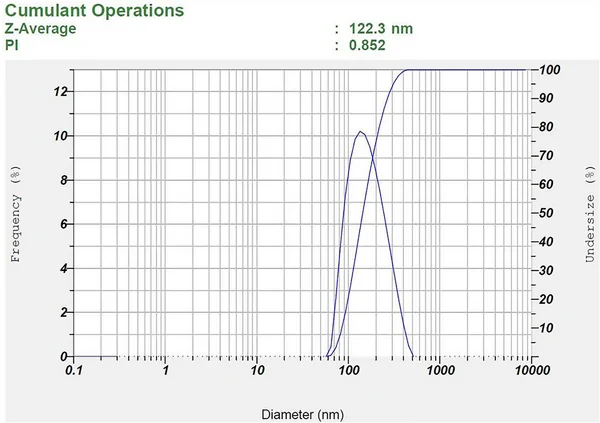
Dynamic light scattering analysis of *Commiphora myrrha* nanoliposomes.

Furthermore, the zeta potential measurement for *C. myrrha* essential oil nanoliposomes indicated a potential of −1.5 mV, suggesting electrostatic stability. This finding further supports the formulation's stability, ensuring its potential effectiveness in medical applications (Figure 2).

These results demonstrate the successful creation of a *C. myrrha* essential oil nanoliposome formulation with desired small dimensions and excellent particle dispersion, laying a solid foundation for its potential therapeutic applications in addressing medical challenges such as leishmaniasis.

### 3.2 Cell Toxicity Results Against *L. tropica* Promastigotes

The results revealed that *C. myrrha* essential oil induced approximately 1.66% ± 0.66% mortality in promastigotes at a concentration of 1 µg/mL, whereas *C. myrrha* essential oil nanoliposomes resulted in 12% ± 1% mortality at the same concentration. With an increase in concentration to 2 µg/mL, *C. myrrha* essential oil and *C. myrrha* essential oil nanoliposomes caused 14% ± 1.15% and 24% ± 0.5% mortality, respectively. At a concentration of 5 µg/mL, *C. myrrha* essential oil led to approximately 24.3% ± 2.02% mortality, whereas *C. myrrha* essential oil nanoliposomes resulted in 34.3% ± 0.88% mortality. Finally, at a concentration of 10 µg/mL, *C. myrrha* essential oil exhibited mortality rates of around 37.66% ± 0.88% in promastigotes, whereas *C. myrrha* essential oil nanoliposomes showed mortality rates of approximately 47.33% ± 0.33%.

**FIGURE 3:**
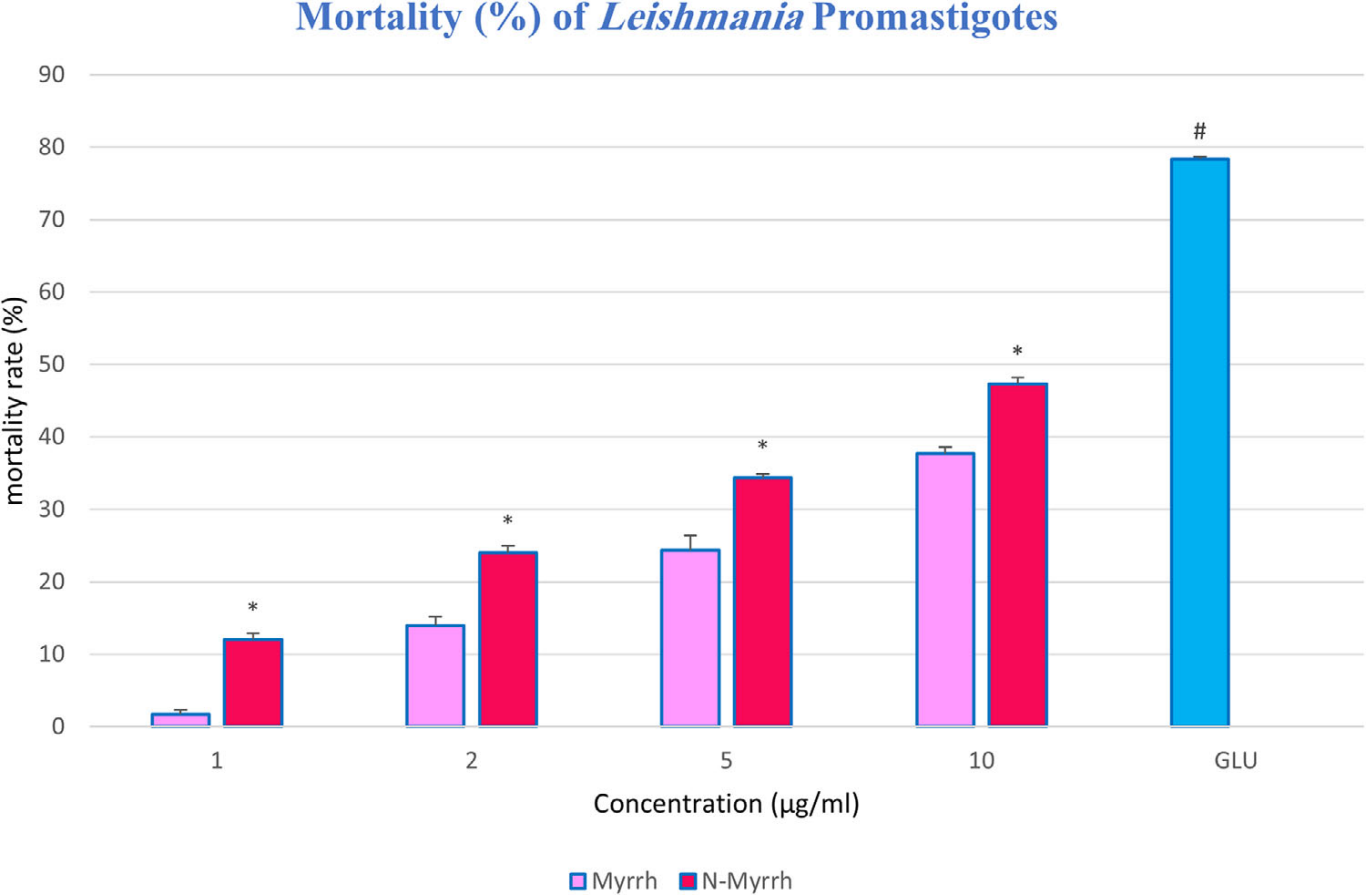
Mortality rate of *Leishmania* promastigotes in MTT assay: comparing treatments with *Commiphora myrrha*, nanoliposomes and the standard drug glucantime (GLU). ‘*’ indicates a significant difference between similar concentrations of *C. myrrha* and *C. myrrha* nanoliposomes. ‘#’ indicates a significant difference between glucantime and other groups. Pink: *C. myrrha*, Red: *C. myrrha* nanoliposomes, Blue: glucantime.

Significant differences in mortality rates were observed between the *C. myrrha* essential oil and *C. myrrha* essential oil nanoliposome formulations across all tested concentrations (*p* < 0.005). Moreover, the mortality rates induced by Glucantime treatment were significantly higher than those induced by *C. myrrha* essential oil and *C. myrrha* essential oil nanoliposomes (*p* < 0.001). These findings underscore the superior efficacy of *C. myrrha* essential oil nanoliposomes compared to *C. myrrha* essential oil in inducing mortality in *L. tropica* promastigotes (Figure 3).

### 3.3 LC50 and LC90 for Myrrh Essential Oil Nanoliposomes

The LC50 and LC90 of *C. myrrha* essential oil were determined using the Pearson goodness of fit test. By converting the concentrations to logarithms and analysing the results with a two‐degree‐of‐freedom test, the study obtained squared chi‐square values of 0.164 and 3.61, leading to *p* values of 0.05 for both cases. These findings indicated the conditions necessary for regression analysis and determining the LC. For *C. myrrha* essential oil, the LC50 was found to be 15.30 µg/mL, with a confidence interval ranging from 10.70 to 28.04 µg/mL at a confidence level of 95%. The LC90 was calculated as 116.36 µg/mL, with a confidence interval from 53.00 to 492.91 µg/mL (Table 1).

**TABLE 1:** Lethal concentrations (LC50 and LC90) for *Commiphora myrrha*.

Confidence limits							
		95% confidence limits for concentration			95% confidence limits for log(concentration)^a^		
	Probability	Estimate	Lower bound	Upper bound	Estimate	Lower bound	Upper bound
Probit	0.010	0.385	0.128	0.705	−0.955	−2.053	−0.349
	0.020	0.593	0.237	0.990	−0.523	−1.442	−0.010
	0.030	0.779	0.348	1.230	−0.249	−1.056	0.207
	0.040	0.958	0.465	1.449	−0.043	−0.767	0.371
	0.050	1.132	0.587	1.659	0.124	−0.533	0.506
	0.060	1.306	0.716	1.862	0.267	−0.335	0.622
	0.070	1.480	0.850	2.064	0.392	−0.162	0.725
	0.080	1.655	0.991	2.265	0.504	−0.009	0.818
	0.090	1.832	1.139	2.467	0.606	0.130	0.903
	0.100	2.013	1.292	2.673	0.699	0.256	0.983
	0.150	2.967	2.144	3.784	1.087	0.763	1.331
	0.200	4.038	3.108	5.146	1.396	1.134	1.638
	0.250	5.261	4.142	6.914	1.660	1.421	1.934
	0.300	6.672	5.231	9.235	1.898	1.655	2.223
	0.350	8.315	6.396	12.263	2.118	1.856	2.507
	0.400	10.247	7.669	16.198	2.327	2.037	2.785
	0.450	12.543	9.091	21.322	2.529	2.207	3.060
	0.500	15.303	10.709	28.047	2.728	2.371	3.334
	0.550	18.671	12.583	36.984	2.927	2.532	3.610
	0.600	22.854	14.797	49.076	3.129	2.694	3.893
	0.650	28.164	17.470	65.835	3.338	2.861	4.187
	0.700	35.100	20.789	89.825	3.558	3.034	4.498
	0.750	44.513	25.057	125.730	3.796	3.221	4.834
	0.800	57.995	30.822	182.997	4.060	3.428	5.209
	0.850	78.945	39.202	283.675	4.369	3.669	5.648
	0.900	116.369	53.004	492.914	4.757	3.970	6.200
	0.910	127.802	57.002	563.352	4.850	4.043	6.334
	0.920	141.500	61.686	651.357	4.952	4.122	6.479
	0.930	158.261	67.278	764.109	5.064	4.209	6.639
	0.940	179.339	74.121	913.310	5.189	4.306	6.817
	0.950	206.825	82.773	1119.422	5.332	4.416	7.021
	0.960	244.546	94.229	1421.867	5.499	4.546	7.260
	0.970	300.475	110.497	1908.069	5.705	4.705	7.554
	0.980	395.105	136.529	2821.389	5.979	4.917	7.945
	0.990	608.304	190.503	5227.881	6.411	5.250	8.562

*Note*: The highlighted rows (Probability = 0.500 and 0.900) in Table represent the LC50 and LC90 values.

a Logarithm base = 2.718.

Similarly, the concentrations were converted to logarithms for *C. myrrha* essential oil nanoliposomes and analysed using a Pearson Fit of Goodness test with two degrees of freedom. Squared chi‐square values of 0.735 and 0.617 were obtained, with corresponding *p* values close to zero for both cases, meeting the requirements for regression analysis and LC determination. The LC50 nanoliposome value was 11.49 µg/mL, with a 95% confidence interval ranging from 7.75 to 23.28 µg/mL. The LC90 for *C. myrrha* essential oil nanoliposomes was determined to be 194.44 µg/mL, with a confidence interval of 67.75–1654.12 µg/mL (Table 2).

**TABLE 2:** Lethal concentrations (LC50 and LC90) for *Commiphora myrrha* nanoliposomes.

Confidence limits							
		95% confidence limits for concentration			95% confidence limits for log(concentration)^a^		
	Probability	Estimate	Lower bound	Upper bound	Estimate	Lower bound	Upper bound
Probit	0.010	0.068	0.008	0.195	−2.693	−4.844	−1.633
	0.020	0.124	0.020	0.307	−2.091	−3.928	−1.181
	0.030	0.181	0.035	0.409	−1.709	−3.348	−0.893
	0.040	0.241	0.054	0.509	−1.422	−2.913	−0.676
	0.050	0.305	0.077	0.607	−1.189	−2.559	−0.499
	0.060	0.372	0.104	0.707	−0.990	−2.259	−0.347
	0.070	0.442	0.136	0.808	−0.815	−1.996	−0.213
	0.080	0.517	0.172	0.911	−0.659	−1.761	−0.093
	0.090	0.596	0.213	1.017	−0.517	−1.548	0.016
	0.100	0.679	0.259	1.125	−0.387	−1.353	0.118
	0.150	1.167	0.575	1.730	0.154	−0.553	0.548
	0.200	1.794	1.066	2.482	0.584	0.064	0.909
	0.250	2.594	1.756	3.486	0.953	0.563	1.249
	0.300	3.612	2.632	4.939	1.284	0.968	1.597
	0.350	4.910	3.655	7.145	1.591	1.296	1.966
	0.400	6.570	4.819	10.510	1.883	1.573	2.352
	0.450	8.709	6.163	15.597	2.164	1.819	2.747
	0.500	11.493	7.755	23.284	2.442	2.048	3.148
	0.550	15.166	9.688	35.016	2.719	2.271	3.556
	0.600	20.103	12.090	53.254	3.001	2.492	3.975
	0.650	26.900	15.151	82.402	3.292	2.718	4.412
	0.700	36.564	19.174	130.845	3.599	2.954	4.874
	0.750	50.924	24.677	215.909	3.930	3.206	5.375
	0.800	73.640	32.631	377.714	4.299	3.485	5.934
	0.850	113.199	45.128	725.949	4.729	3.810	6.587
	0.900	194.442	67.759	1654.121	5.270	4.216	7.411
	0.910	221.584	74.735	2018.522	5.401	4.314	7.610
	0.920	255.379	83.124	2506.087	5.543	4.420	7.826
	0.930	298.518	93.432	3179.396	5.699	4.537	8.064
	0.940	355.366	106.455	4147.664	5.873	4.668	8.330
	0.950	433.529	123.525	5617.273	6.072	4.816	8.634
	0.960	547.594	147.092	8022.877	6.306	4.991	8.990
	0.970	729.739	182.288	12,436.691	6.593	5.206	9.428
	0.980	1068.922	242.393	22,277.389	6.974	5.491	10.011
	0.990	1950.892	379.679	55,852.306	7.576	5.939	10.930

*Note*: The highlighted rows (Probability = 0.500 and 0.900) in Table represent the LC50 and LC90 values.

a Logarithm base = 2.718.

## 4 Discussion

With the rise in drug resistance and the prevalence of side effects associated with conventional medications, researchers are increasingly turning their attention to natural alternatives for treating infections (AlSheikh et al. 2020; Ghafouri et al. 2023). Essential oils, plant extracts, and other plant‐derived compounds have emerged as promising resources in the search for drugs to combat diseases like leishmaniasis (Cortes et al. 2020; Passero et al. 2018). These substances, including alkaloids, terpenes, flavonoids, benzopyrans, phenolics and sesquiterpene lactones, have demonstrated leishmanial properties across various plant species (Oryan 2015; Raj et al. 2020).

For instance, terpenoid compounds such as β‐caryophyllene and nerolidol have exhibited anti‐*Leishmania* effects, possibly by inhibiting cellular isoprenoid biosynthesis (Moura do Carmo et al. 2012). Additionally, lipid constituents present in essential oils have been shown to impact the plasma membranes of *Leishmania* promastigotes, leading to cell rupture and release of large molecules (Hughes et al. 2023; Karampetsou et al. 2021). Research by Carvalho et al. (2017) highlighted the antileishmanial activity of ‘sweet basil’ against clinically relevant intracellular amastigote forms while demonstrating minimal toxicity to host cells (Carvalho et al. 2017). Similarly, studies by Ramos et al. (2014) and Sifaoui et al. (2014) revealed potent lethal effects of essential oils and triterpenic acids on *Leishmania* promastigotes, respectively (Ramos et al. 2014; Sifaoui et al. 2014). Furthermore, investigations into using nanoparticles such as gold and silver have shown promising results in combating single‐celled organisms like *Leishmania* (Elmi et al. 2013).

Nanocoating has emerged as one of the most innovative technologies in the fight against parasites (Meral et al. 2022). It offers transformative benefits in various fields, such as the enhancement of antibiotic properties and the ability to enhance plant extract efficacy. Due to the unique properties of these coatings, which are typically composed of nanoscale materials, these coatings come with numerous advantages over conventional coatings (Prasad et al. 2021). It has many capabilities, including increased surface area, enhanced reactivity and the capacity to release products at a controlled rate (Mercan et al. 2022). Antiparasitic drugs can be delivered more efficiently and effectively with nanocoatings when providing therapeutic agents in larger quantities (Harish et al. 2022). Using these coatings, active compounds can be protected from degradation by encapsulating them in nanocarriers, such as nanoparticles or nanocapsules, which then carry the drugs when administered. In addition to improving their solubility, they can enhance their ability to penetrate biological barriers and make them more soluble. This method enhances medications’ bioavailability and stability, ensuring sustained release (Erdoğar et al. 2018). Consequently, they are capable of fighting parasites better because of this. For example, nanocoatings can improve antibiotic absorption and resistance in antiparasitic medications, making them more effective and reliable treatments. This can provide a better choice for those susceptible to poor drug absorption and resistance.

Further, nanocoatings have proven to be beneficial in amplifying the effects of plant extracts and are widely used in the pharmaceutical industry due to their therapeutic properties (Ansari 2023; Detsi et al. 2020; Petrovic et al. 2024). Often, plant extracts are very rich in bioactive compounds; therefore, nanotechnology can be a great tool for enhancing their stability, solubility and targeted delivery of these compounds (Kyriakoudi et al. 2021; Rachmawati et al. 2020; Wahab et al. 2022). These extracts can be encapsulated with nanocoatings, protecting them from environmental factors such as light and oxygen (Singh et al. 2017). This could potentially degrade their active ingredients when exposed. The extracts are much more bioavailable to the body through encapsulation, so their absorption and utilization are improved since they can be used more efficiently (Gunasekaran et al. 2014; Puglia et al. 2017). Moreover, nanocoatings can also facilitate the controlled release of compounds derived from plants. This will ensure that these compounds are delivered sustainably to the site of action of the compound. The benefits of this targeted delivery method are that, while maximizing the therapeutic potential of the extracts, it also minimizes the likelihood of adverse effects, as it reduces the number of active molecules entering the systemic circulation.

The present study explored the nanoliposomes containing *C. myrrha* essential oil in combating *L. tropica*. The study's findings indicate that *C. myrrha* essential oil nanoliposomes positively impacted *L. tropica* promastigotes, suggesting their potential as a therapeutic approach for combating leishmaniasis. However, further validation of these findings in animal settings and toxicity assessment is essential. Additionally, exploring the efficacy of plant compounds against *L. tropica* parasites under laboratory and real‐life conditions is recommended to develop practical therapeutic approaches using natural compounds. This holistic approach will provide valuable insights into utilizing plant‐based treatments for leishmaniasis, contributing to the development of effective therapeutic strategies.

The current study had several limitations, notably the absence of comprehensive assessments such as drug release studies and drug encapsulation tests and detailed analyses like scanning electron microscopy (SEM) and transmission electron microscopy (TEM). These additional tests could significantly enhance our understanding of the nanocapsules’ properties, including their release kinetics and encapsulation efficiency. Incorporating these techniques in future research could provide deeper insights into the structural and functional characteristics of the nanocapsules, which are crucial for optimizing their performance. Furthermore, conducting in vivo studies is highly recommended, as they would offer valuable information on the nanocapsules’ biological interactions and overall efficacy, providing a more complete picture of their potential applications and benefits.

Additionally, medicinal products should be combined with conventional drugs such as Glucantime. These components are a part of complementary medicine, potentiate the immune response, and can completely alleviate the organisms. No drugs alone could be a miracle. The drug combination is now a routine strategy for treating leishmaniasis. Further advanced studies are crucial to assess the in vivo potential of myrrh alone and in combination with Glucantime.

## 5 Conclusion

In conclusion, the in vitro investigations conducted on *C. myrrha* essential oil nanoliposomes have demonstrated promising results in eliminating *L. tropica*. The effectiveness of nanoliposomes containing *C. myrrha* essential oil suggests their potential as an alternative or adjunctive treatment for leishmaniasis, complementing existing synthetic medications. The beneficial impact observed in these experiments highlights the therapeutic potential of natural compounds encapsulated in nanoliposomal formulations. By harnessing the properties of myrrh essential oil and leveraging the advantages of nanotechnology, such as targeted drug delivery and enhanced bioavailability, these nanoliposomes offer a promising avenue for combating leishmaniasis effectively.

## Author Contributions

**Zahra Akbari**: writing – original draft, writing – review and editing, methodology, software, formal analysis, data curation. **Mohammad Reza Youssefi**: writing – original draft, funding acquisition, conceptualization, investigation, methodology, validation, visualization, writing – review and editing, project administration, formal analysis, software, data curation, supervision, resources. **Mohaddeseh Abouhosseini Tabari**: data curation, formal analysis, writing – review and editing, writing – original draft, investigation, methodology.

## Funding

The authors have nothing to report.

## Ethics Statement

All applicable international, national, and/or institutional guidelines for the care and use of animals were followed. IR.IAU.BABOL.REC.1401.128.

## Conflicts of Interest

The authors declare no conflicts of interest.

## Data Availability

The datasets generated during and/or analysed during the current study are available from the corresponding author upon reasonable request.
